# A second monoclinic polymorph of 4-(2-hydr­oxy-4-methoxy­benzyl­ideneamino)-1,5-dimethyl-2-phenyl-1*H*-pyrazol-3(2*H*)-one

**DOI:** 10.1107/S1600536808029498

**Published:** 2008-09-20

**Authors:** Zhao-Fu Zhu, Xi-Hai Shen, Xiao-Guang Tang

**Affiliations:** a Department of Chemistry, Hebei Normal College of Science and Technology, Qinhuangdao 066600, People’s Republic of China

## Abstract

The title compound, C_19_H_19_N_3_O_3_, prepared by condensing 4-amino­anti­pyrine and 4-meth­oxy-2-hydroxy­benzaldehyde in methanol, is the second monoclinic polymorph of this compound which crystallizes in the space group *C*2/*c*. The structure was previously reported [Wang, Zhang, Yan, Zheng & Yang (2007 ▶). *Acta Cryst*. E**63**, o1245–o1246] in the space group *P*2_1_/*c*. The hydroxyl group is disordered over two positions with occupancies of 0.787 (4) and 0.213 (4). The triply substituted benzene ring and the phenyl ring form dihedral angles of 12.2 (2) and 53.7 (2)°, respectively, with the pyrazolone ring; the corresponding values in the *P*2_1_/*c* polymorph are 7.5 (2) and 42.6 (2)°. Intra­molecular O—H⋯N and C—H⋯O hydrogen bonds are observed in the major disorder component. Adjacent molecules are linked through intermolecular O—H⋯O hydrogen bonds, forming dimers.

## Related literature

For the *P*2_1_/*c* polymorph of the title compound, see: Wang *et al.* (2007 ▶). For related structures, see: Duan *et al.* (2006 ▶); Jing *et al.* (2006 ▶); Sun *et al.* (2006 ▶); Wen (2005 ▶); Zhang *et al.* (2007 ▶); Zheng *et al.* (2006 ▶).
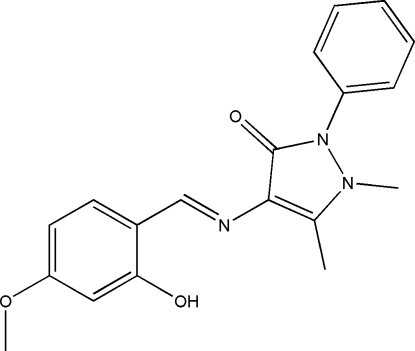


## Experimental

### 

#### Crystal data

C_19_H_19_N_3_O_3_
*M* *_r_* = 337.37
Monoclinic, 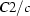

*a* = 30.581 (3) Å
*b* = 6.906 (2) Å
*c* = 17.059 (3) Å
β = 110.500 (2)°
*V* = 3374.6 (12) Å^3^
*Z* = 8
Mo *K*α radiation
μ = 0.09 mm^−1^
*T* = 298 (2) K
0.30 × 0.28 × 0.27 mm

#### Data collection

Bruker SMART CCD area-detector diffractometer
Absorption correction: multi-scan (*SADABS*; Bruker, 2001 ▶) *T*_min_ = 0.973, *T*_max_ = 0.976
9315 measured reflections
3634 independent reflections
2355 reflections with *I* > 2σ(*I*)
*R*_int_ = 0.037

#### Refinement

*R*[*F* ^2^ > 2σ(*F* ^2^)] = 0.048
*wR*(*F* ^2^) = 0.145
*S* = 1.01
3634 reflections
241 parameters
H-atom parameters constrained
Δρ_max_ = 0.19 e Å^−3^
Δρ_min_ = −0.20 e Å^−3^

### 

Data collection: *SMART* (Bruker, 2007 ▶); cell refinement: *SAINT* (Bruker, 2007 ▶); data reduction: *SAINT*; program(s) used to solve structure: *SHELXTL* (Sheldrick, 2008 ▶); program(s) used to refine structure: *SHELXTL*; molecular graphics: *SHELXTL*; software used to prepare material for publication: *SHELXTL*.

## Supplementary Material

Crystal structure: contains datablocks global, I. DOI: 10.1107/S1600536808029498/ci2671sup1.cif

Structure factors: contains datablocks I. DOI: 10.1107/S1600536808029498/ci2671Isup2.hkl

Additional supplementary materials:  crystallographic information; 3D view; checkCIF report

## Figures and Tables

**Table 1:** Hydrogen-bond geometry (Å, °)

*D*—H⋯*A*	*D*—H	H⋯*A*	*D*⋯*A*	*D*—H⋯*A*
O1—H1⋯N1	0.82	1.88	2.605 (2)	147
O1′—H1′⋯O3^i^	0.82	1.80	2.609 (6)	169
C7—H7⋯O3	0.93	2.33	3.006 (5)	129

Symmetry code: (i) 
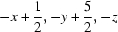
.

**Fig. 1.**
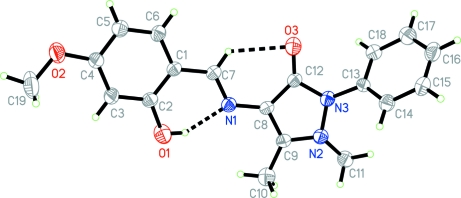
The molecular structure of the title compound, showing 30% probability displacement ellisoids. Only the major disorder component is shown. Intramolecular hydrogen bonds are shown as dashed lines.

## supplementary crystallographic information

### Comment

The crystal structure of the title compound has been previously reported by Wang *et al.* (2007) in the monoclinic space group *P*2_1_/*c*. We report here the structure of the second monoclinic polymorph of the title compound, in the space group *C*2/*c*.

In the title molecule (Fig. 1), the pyrazolone ring makes dihedral angles of 12.2 (2) and 53.7 (2)°, respectively, with the triply substituted C1–C6 benzene ring and the unsubstituted C13—C18 benzene ring; the corresponding values in the *P*2_1_/*c* polymorph are 7.5 (2) and 42.6 (2)°. The bond lengths and angles are within normal ranges and comparable with those in related similar compounds (Duan *et al.*, 2006; Jing *et al.*, 2006; Zheng *et al.*, 2006; Sun *et al.*, 2006; Zhang *et al.*, 2007; Wen, 2005). Intramolecular O—H···N and C—H···O hydrogen bonds (Table 1) are observed in the molecular structure.

### Experimental

4-Methoxy-2-hydroxybenzaldehyde (152.1 mg, 1.0 mmol) and 4-aminoantipyrine (203.2 mg, 1.0 mmol) were added in methanol (60 ml). The mixture was refluxed for 30 min, then cooled to room temperature, yielding colourless solution. Colourless single crystals were formed when the solution was evaporated in air for several days.

### Refinement

The hydroxyl group is disordered over two positions with refined occupancies of 0.787 (4) and 0.213 (4). H atoms were placed in idealized positions and constrained to ride on their parent atoms, with C–H distances of 0.93–0.96 Å, O–H distance of 0.82 Å, and with *U*_iso_(H) set at 1.2*U*_eq_(C) and 1.5*U*_eq_(O and methyl C).

### Crystal data

**Table:**

C_19_H_19_N_3_O_3_	*F*(000) = 1424
*M**_r_* = 337.37	*D*_x_ = 1.328 Mg m^−^^3^
Monoclinic, *C*2/*c*	Mo *K*α radiation, λ = 0.71073 Å
Hall symbol: -C 2yc	Cell parameters from 2554 reflections
*a* = 30.581 (3) Å	θ = 2.4–25.6°
*b* = 6.906 (2) Å	µ = 0.09 mm^−^^1^
*c* = 17.059 (3) Å	*T* = 298 K
β = 110.500 (2)°	Block, colourless
*V* = 3374.6 (12) Å^3^	0.30 × 0.28 × 0.27 mm
*Z* = 8	

### Data collection

**Table:**

Bruker SMART CCD area-detector diffractometer	3634 independent reflections
Radiation source: fine-focus sealed tube	2355 reflections with *I* > 2σ(*I*)
graphite	*R*_int_ = 0.037
ω scans	θ_max_ = 27.0°, θ_min_ = 1.4°
Absorption correction: multi-scan (SADABS; Bruker, 2001)	*h* = −36→38
*T*_min_ = 0.973, *T*_max_ = 0.976	*k* = −8→8
9315 measured reflections	*l* = −20→21

### Refinement

**Table:**

Refinement on *F*^2^	Primary atom site location: structure-invariant direct methods
Least-squares matrix: full	Secondary atom site location: difference Fourier map
*R*[*F*^2^ > 2σ(*F*^2^)] = 0.048	Hydrogen site location: inferred from neighbouring sites
*wR*(*F*^2^) = 0.145	H-atom parameters constrained
*S* = 1.02	*w* = 1/[σ^2^(*F*_o_^2^) + (0.071*P*)^2^ + 0.6977*P*] where *P* = (*F*_o_^2^ + 2*F*_c_^2^)/3
3634 reflections	(Δ/σ)_max_ = 0.001
241 parameters	Δρ_max_ = 0.19 e Å^−^^3^
0 restraints	Δρ_min_ = −0.20 e Å^−^^3^

### Special details

**Table:**

Geometry. All e.s.d.'s (except the e.s.d. in the dihedral angle between two l.s. planes) are estimated using the full covariance matrix. The cell e.s.d.'s are taken into account individually in the estimation of e.s.d.'s in distances, angles and torsion angles; correlations between e.s.d.'s in cell parameters are only used when they are defined by crystal symmetry. An approximate (isotropic) treatment of cell e.s.d.'s is used for estimating e.s.d.'s involving l.s. planes.
Refinement. Refinement of *F*^2^ against ALL reflections. The weighted *R*-factor *wR* and goodness of fit *S* are based on *F*^2^, conventional *R*-factors *R* are based on *F*, with *F* set to zero for negative *F*^2^. The threshold expression of *F*^2^ > σ(*F*^2^) is used only for calculating *R*-factors(gt) *etc*. and is not relevant to the choice of reflections for refinement. *R*-factors based on *F*^2^ are statistically about twice as large as those based on *F*, and *R*- factors based on ALL data will be even larger.

### Fractional atomic coordinates and isotropic or equivalent isotropic displacement parameters (Å^2^)

**Table:**

	*x*	*y*	*z*	*U*_iso_*/*U*_eq_	Occ. (<1)
O1	0.13337 (6)	0.6567 (2)	0.02029 (14)	0.0720 (7)	0.787 (4)
H1	0.1613	0.6337	0.0427	0.108*	0.787 (4)
O1'	0.1955 (2)	1.2396 (10)	−0.0333 (4)	0.071 (3)	0.213 (4)
H1'	0.1891	1.3241	−0.0693	0.106*	0.213 (4)
O2	0.03260 (5)	1.1507 (2)	−0.13459 (11)	0.0834 (5)	
O3	0.31552 (5)	0.96750 (18)	0.13504 (9)	0.0628 (4)	
N1	0.22230 (5)	0.7333 (2)	0.08331 (9)	0.0483 (4)	
N2	0.32298 (5)	0.5274 (2)	0.23447 (9)	0.0494 (4)	
N3	0.34320 (5)	0.6934 (2)	0.21511 (9)	0.0491 (4)	
C1	0.16608 (6)	0.9540 (2)	−0.00342 (11)	0.0450 (4)	
C2	0.12777 (7)	0.8350 (3)	−0.01178 (12)	0.0505 (5)	
H2	0.1327	0.7125	0.0124	0.061*	0.213 (4)
C3	0.08307 (7)	0.8956 (3)	−0.05505 (13)	0.0586 (5)	
H3	0.0580	0.8144	−0.0601	0.070*	
C4	0.07548 (7)	1.0762 (3)	−0.09092 (13)	0.0573 (5)	
C5	0.11288 (7)	1.1968 (3)	−0.08392 (12)	0.0586 (5)	
H5	0.1078	1.3190	−0.1084	0.070*	
C6	0.15705 (6)	1.1355 (3)	−0.04112 (12)	0.0511 (5)	
H6	0.1820	1.2171	−0.0369	0.061*	0.787 (4)
C7	0.21326 (6)	0.8961 (2)	0.04376 (11)	0.0468 (4)	
H7	0.2378	0.9780	0.0458	0.056*	
C8	0.26761 (6)	0.6861 (2)	0.13421 (10)	0.0437 (4)	
C9	0.27825 (6)	0.5207 (2)	0.18071 (11)	0.0458 (4)	
C10	0.24772 (7)	0.3533 (3)	0.17836 (14)	0.0658 (6)	
H10A	0.2179	0.3725	0.1351	0.099*	
H10B	0.2436	0.3411	0.2314	0.099*	
H10C	0.2618	0.2375	0.1670	0.099*	
C11	0.35235 (7)	0.3601 (3)	0.26802 (13)	0.0625 (6)	
H11A	0.3354	0.2677	0.2883	0.094*	
H11B	0.3798	0.4001	0.3132	0.094*	
H11C	0.3613	0.3019	0.2248	0.094*	
C12	0.30866 (6)	0.8026 (2)	0.15627 (11)	0.0468 (4)	
C13	0.38321 (6)	0.7784 (2)	0.27470 (11)	0.0460 (4)	
C14	0.39069 (7)	0.7743 (3)	0.35949 (12)	0.0559 (5)	
H14	0.3703	0.7077	0.3795	0.067*	
C15	0.42859 (8)	0.8700 (3)	0.41369 (14)	0.0673 (6)	
H15	0.4341	0.8665	0.4709	0.081*	
C16	0.45828 (8)	0.9701 (3)	0.38483 (15)	0.0717 (6)	
H16	0.4836	1.0358	0.4221	0.086*	
C17	0.45062 (7)	0.9736 (3)	0.30043 (15)	0.0672 (6)	
H17	0.4708	1.0424	0.2807	0.081*	
C18	0.41342 (7)	0.8764 (3)	0.24504 (13)	0.0545 (5)	
H18	0.4087	0.8768	0.1881	0.065*	
C19	−0.00684 (9)	1.0308 (4)	−0.1448 (3)	0.1287 (14)	
H19A	−0.0037	0.9120	−0.1715	0.193*	
H19B	−0.0347	1.0966	−0.1788	0.193*	
H19C	−0.0088	1.0030	−0.0910	0.193*	

### Atomic displacement parameters (Å^2^)

**Table:**

	*U*^11^	*U*^22^	*U*^33^	*U*^12^	*U*^13^	*U*^23^
O1	0.0605 (11)	0.0448 (11)	0.1057 (17)	−0.0043 (9)	0.0229 (11)	0.0205 (10)
O1'	0.056 (4)	0.064 (4)	0.079 (5)	−0.012 (3)	0.005 (3)	0.032 (4)
O2	0.0526 (8)	0.0619 (9)	0.1159 (14)	0.0025 (7)	0.0047 (8)	0.0090 (9)
O3	0.0643 (8)	0.0470 (8)	0.0663 (9)	−0.0092 (7)	0.0094 (7)	0.0151 (6)
N1	0.0518 (9)	0.0447 (8)	0.0470 (9)	−0.0007 (7)	0.0155 (7)	0.0007 (7)
N2	0.0561 (9)	0.0376 (8)	0.0509 (9)	−0.0017 (7)	0.0142 (8)	0.0053 (7)
N3	0.0541 (9)	0.0402 (8)	0.0483 (9)	−0.0070 (7)	0.0119 (7)	0.0041 (7)
C1	0.0505 (10)	0.0432 (9)	0.0406 (10)	−0.0025 (8)	0.0150 (8)	−0.0014 (8)
C2	0.0557 (11)	0.0404 (10)	0.0556 (11)	−0.0029 (8)	0.0197 (9)	0.0005 (8)
C3	0.0488 (11)	0.0479 (11)	0.0750 (14)	−0.0069 (9)	0.0167 (10)	−0.0035 (10)
C4	0.0524 (11)	0.0494 (11)	0.0630 (13)	0.0029 (9)	0.0114 (10)	−0.0009 (9)
C5	0.0618 (12)	0.0461 (11)	0.0608 (13)	−0.0010 (9)	0.0125 (10)	0.0087 (9)
C6	0.0530 (11)	0.0479 (10)	0.0497 (11)	−0.0073 (9)	0.0147 (9)	0.0031 (8)
C7	0.0526 (10)	0.0454 (10)	0.0425 (10)	−0.0047 (8)	0.0167 (8)	−0.0021 (8)
C8	0.0513 (10)	0.0394 (9)	0.0404 (9)	−0.0015 (8)	0.0162 (8)	−0.0020 (7)
C9	0.0541 (10)	0.0410 (9)	0.0442 (10)	−0.0015 (8)	0.0195 (9)	−0.0011 (8)
C10	0.0708 (13)	0.0508 (11)	0.0740 (14)	−0.0116 (10)	0.0229 (12)	0.0091 (10)
C11	0.0688 (13)	0.0471 (11)	0.0632 (13)	0.0063 (10)	0.0127 (11)	0.0108 (9)
C12	0.0562 (11)	0.0402 (9)	0.0413 (10)	−0.0026 (8)	0.0138 (9)	0.0011 (8)
C13	0.0453 (10)	0.0397 (9)	0.0487 (11)	0.0024 (8)	0.0111 (9)	−0.0004 (8)
C14	0.0554 (11)	0.0612 (12)	0.0505 (11)	−0.0039 (10)	0.0180 (10)	0.0004 (9)
C15	0.0663 (13)	0.0772 (14)	0.0496 (12)	−0.0062 (12)	0.0091 (11)	−0.0061 (10)
C16	0.0600 (13)	0.0742 (15)	0.0689 (15)	−0.0139 (11)	0.0075 (11)	−0.0059 (12)
C17	0.0565 (12)	0.0623 (13)	0.0838 (16)	−0.0101 (10)	0.0255 (12)	0.0009 (12)
C18	0.0590 (11)	0.0527 (11)	0.0536 (11)	−0.0026 (9)	0.0223 (10)	0.0005 (9)
C19	0.0503 (14)	0.087 (2)	0.211 (4)	−0.0084 (14)	−0.0024 (18)	0.023 (2)

### Geometric parameters (Å, °)

**Table:**

O1—C2	1.333 (2)	C7—H7	0.93
O1—H1	0.82	C8—C9	1.363 (2)
O1'—C6	1.345 (6)	C8—C12	1.426 (2)
O1'—H1'	0.82	C9—C10	1.478 (2)
O2—C4	1.362 (2)	C10—H10A	0.96
O2—C19	1.422 (3)	C10—H10B	0.96
O3—C12	1.235 (2)	C10—H10C	0.96
N1—C7	1.291 (2)	C11—H11A	0.96
N1—C8	1.392 (2)	C11—H11B	0.96
N2—C9	1.355 (2)	C11—H11C	0.96
N2—N3	1.3959 (19)	C13—C18	1.376 (3)
N2—C11	1.452 (2)	C13—C14	1.383 (3)
N3—C12	1.396 (2)	C14—C15	1.373 (3)
N3—C13	1.416 (2)	C14—H14	0.93
C1—C6	1.392 (2)	C15—C16	1.363 (3)
C1—C2	1.397 (2)	C15—H15	0.93
C1—C7	1.441 (2)	C16—C17	1.376 (3)
C2—C3	1.372 (3)	C16—H16	0.93
C2—H2	0.93	C17—C18	1.373 (3)
C3—C4	1.373 (3)	C17—H17	0.93
C3—H3	0.93	C18—H18	0.93
C4—C5	1.386 (3)	C19—H19A	0.96
C5—C6	1.359 (3)	C19—H19B	0.96
C5—H5	0.93	C19—H19C	0.96
C6—H6	0.93		
			
C2—O1—H1	109.5	C8—C9—C10	128.31 (17)
C6—O1'—H1'	109.5	C9—C10—H10A	109.5
C4—O2—C19	117.40 (17)	C9—C10—H10B	109.5
C7—N1—C8	120.96 (15)	H10A—C10—H10B	109.5
C9—N2—N3	107.07 (13)	C9—C10—H10C	109.5
C9—N2—C11	125.34 (14)	H10A—C10—H10C	109.5
N3—N2—C11	118.86 (14)	H10B—C10—H10C	109.5
N2—N3—C12	109.12 (13)	N2—C11—H11A	109.5
N2—N3—C13	120.92 (14)	N2—C11—H11B	109.5
C12—N3—C13	122.55 (14)	H11A—C11—H11B	109.5
C6—C1—C2	117.34 (16)	N2—C11—H11C	109.5
C6—C1—C7	120.37 (16)	H11A—C11—H11C	109.5
C2—C1—C7	122.27 (16)	H11B—C11—H11C	109.5
O1—C2—C3	117.58 (18)	O3—C12—N3	123.06 (16)
O1—C2—C1	121.24 (18)	O3—C12—C8	131.86 (17)
C3—C2—C1	121.16 (17)	N3—C12—C8	105.01 (14)
C3—C2—H2	119.4	C18—C13—C14	120.51 (17)
C1—C2—H2	119.4	C18—C13—N3	117.56 (17)
C2—C3—C4	119.87 (18)	C14—C13—N3	121.82 (17)
C2—C3—H3	120.1	C15—C14—C13	119.09 (19)
C4—C3—H3	120.1	C15—C14—H14	120.5
O2—C4—C3	124.53 (18)	C13—C14—H14	120.5
O2—C4—C5	115.33 (17)	C16—C15—C14	120.8 (2)
C3—C4—C5	120.14 (18)	C16—C15—H15	119.6
C6—C5—C4	119.62 (17)	C14—C15—H15	119.6
C6—C5—H5	120.2	C15—C16—C17	119.7 (2)
C4—C5—H5	120.2	C15—C16—H16	120.1
O1'—C6—C5	123.9 (3)	C17—C16—H16	120.1
O1'—C6—C1	114.2 (3)	C18—C17—C16	120.5 (2)
C5—C6—C1	121.86 (17)	C18—C17—H17	119.7
C5—C6—H6	119.1	C16—C17—H17	119.7
C1—C6—H6	119.1	C17—C18—C13	119.2 (2)
N1—C7—C1	121.53 (17)	C17—C18—H18	120.4
N1—C7—H7	119.2	C13—C18—H18	120.4
C1—C7—H7	119.2	O2—C19—H19A	109.5
C9—C8—N1	122.81 (16)	O2—C19—H19B	109.5
C9—C8—C12	108.09 (15)	H19A—C19—H19B	109.5
N1—C8—C12	128.62 (15)	O2—C19—H19C	109.5
N2—C9—C8	110.13 (15)	H19A—C19—H19C	109.5
N2—C9—C10	121.55 (16)	H19B—C19—H19C	109.5
			
C9—N2—N3—C12	7.87 (18)	C11—N2—C9—C8	−153.47 (18)
C11—N2—N3—C12	157.19 (16)	N3—N2—C9—C10	174.23 (17)
C9—N2—N3—C13	158.51 (16)	C11—N2—C9—C10	27.5 (3)
C11—N2—N3—C13	−52.2 (2)	N1—C8—C9—N2	−169.67 (15)
C6—C1—C2—O1	178.03 (19)	C12—C8—C9—N2	3.0 (2)
C7—C1—C2—O1	−3.5 (3)	N1—C8—C9—C10	9.3 (3)
C6—C1—C2—C3	−0.4 (3)	C12—C8—C9—C10	−177.97 (18)
C7—C1—C2—C3	178.12 (18)	N2—N3—C12—O3	171.32 (17)
O1—C2—C3—C4	−178.5 (2)	C13—N3—C12—O3	21.3 (3)
C1—C2—C3—C4	−0.1 (3)	N2—N3—C12—C8	−5.92 (18)
C19—O2—C4—C3	−1.2 (4)	C13—N3—C12—C8	−155.98 (16)
C19—O2—C4—C5	178.8 (2)	C9—C8—C12—O3	−175.1 (2)
C2—C3—C4—O2	−179.54 (19)	N1—C8—C12—O3	−2.9 (3)
C2—C3—C4—C5	0.4 (3)	C9—C8—C12—N3	1.84 (19)
O2—C4—C5—C6	179.70 (19)	N1—C8—C12—N3	174.00 (16)
C3—C4—C5—C6	−0.2 (3)	N2—N3—C13—C18	150.64 (16)
C4—C5—C6—O1'	177.8 (5)	C12—N3—C13—C18	−62.7 (2)
C4—C5—C6—C1	−0.2 (3)	N2—N3—C13—C14	−33.2 (2)
C2—C1—C6—O1'	−177.6 (4)	C12—N3—C13—C14	113.5 (2)
C7—C1—C6—O1'	3.8 (5)	C18—C13—C14—C15	0.2 (3)
C2—C1—C6—C5	0.6 (3)	N3—C13—C14—C15	−175.85 (18)
C7—C1—C6—C5	−177.99 (18)	C13—C14—C15—C16	0.8 (3)
C8—N1—C7—C1	−174.67 (15)	C14—C15—C16—C17	−0.8 (3)
C6—C1—C7—N1	175.94 (17)	C15—C16—C17—C18	−0.3 (3)
C2—C1—C7—N1	−2.5 (3)	C16—C17—C18—C13	1.3 (3)
C7—N1—C8—C9	176.18 (17)	C14—C13—C18—C17	−1.3 (3)
C7—N1—C8—C12	5.1 (3)	N3—C13—C18—C17	174.94 (18)
N3—N2—C9—C8	−6.7 (2)		

### Hydrogen-bond geometry (Å, °)

**Table:**

*D*—H···*A*	*D*—H	H···*A*	*D*···*A*	*D*—H···*A*
O1—H1···N1	0.82	1.88	2.605 (2)	147
O1'—H1'···O3^i^	0.82	1.80	2.609 (6)	169
C7—H7···O3	0.93	2.33	3.006 (5)	129

Symmetry codes: (i) −*x*+1/2, −*y*+5/2, −*z*.
